# Continued in vitro cefazolin susceptibility in methicillin-susceptible *Staphylococcus aureus*

**DOI:** 10.1186/s12941-018-0257-x

**Published:** 2018-02-20

**Authors:** Benjamin H. Gern, Alexander L. Greninger, Scott J. Weissman, Jennifer R. Stapp, Yue Tao, Xuan Qin

**Affiliations:** 1 Department of Pediatrics, Seattle Children’s Hospital, University of Washington, Seattle, WA USA; 2 Seattle Children’s Microbiology Laboratory, Seattle, USA; 3 Department of Laboratory Medicine, University of Washington, Seattle, WA USA; 4 Present Address: Shanghai Children’s Medical Center, Translational Research Institute, Shanghai Jiao Tong University School of Medicine, Shanghai, 200127 China

**Keywords:** *Staphylococcus aureus*, MSSA, Cefazolin, Ceftriaxone, MIC

## Abstract

**Objectives:**

In vitro trends of cefazolin and ceftriaxone susceptibilities from pediatric clinical isolates of methicillin-susceptible *Staphylococcus aureus* (MSSA) between 2011 and 2016 were analyzed for surveillance.

**Methods:**

Our laboratory continues to use agar disk diffusion for staphylococcal susceptibilities applying Clinical Laboratory Standard Institute’s 2012 breakpoints.

**Results:**

A total of 3992 MSSA clinical isolates in the last 6 years were analyzed for their in vitro cefazolin and ceftriaxone susceptibilities. While all MSSA isolates exhibited cefazolin susceptibilities within the “susceptible” zone range, there have been a proportion of isolates with ceftriaxone susceptibilities falling in “intermediate” zones, ranging from 2.6% in 2011 to 8.3% in 2016.

**Conclusions:**

Cefazolin continues to be the recommended agent for MSSA treatment at our institution, reflected by the finding that only 2% (6/321) of patients who received ceftriaxone as definitive therapy for MSSA bacteremia during the study period. We have confirmed the cefoxitin-predicted MSSA susceptibility to cefazolin, but have found concerning drifts in ceftriaxone susceptibilities by continued in vitro monitoring over the last 6 years.

**Electronic supplementary material:**

The online version of this article (10.1186/s12941-018-0257-x) contains supplementary material, which is available to authorized users.

## Background

The development of resistance of *Staphylococcus aureus* to β-lactam antibiotics has been well-characterized starting from the first uses of penicillin [1]. This first occurred starting with production of penicillinase, then *mecA*- and *vanA*-determined mechanisms to alter the components of cell wall synthesis [2, 3]. The gene *mecA* confers the majority of resistance to penicillinase-stable *β*-lactams [4]. For over 20 years, the Clinical Laboratory Standard Institute (CLSI) has included all β-lactam agents, including all classes of cephalosporins against staphylococcal species, with minimum inhibitory concentration (MIC) or zone size breakpoint recommendations [5]. In January 2013, CLSI eliminated all *β*-lactam antibiotic breakpoints for methicillin-susceptible *S. aureus* (MSSA), except oxacillin, cefoxitin, penicillin, and ceftaroline [6]. This recommendation derives from the understanding that susceptibility to antistaphylococcal *β*-lactams can be inferred using the above agents. Following this recommendation, there have been a few small in vitro and clinical studies that have examined the question of “inferred susceptibility” for MSSA [7–10]. This study intends to use our existing in vitro susceptibility data to inform future practices.

## Methods

The agar disk diffusion method has been consistently used in our laboratory for susceptibility testing of all *Staphylococcus* spp. for more than 20 years. The susceptibility panel included antibiotic disks (Remel, USA): penicillin (10 units), oxacillin (1 μg, prior to 2011) or cefoxitin (30 μg after 2011), amoxicillin/clavulanate (20/10 μg), cefazolin (10 μg), ceftriaxone (10 μg), meropenem (10 μg), gentamicin (10 μg), erythromycin (15 μg), clindamycin (2 μg), ciprofloxacin (5 μg), rifampin (5 μg), sulfamethoxazole-trimethoprim (SMX-TMP, 1.25/23.75 μg), linezolid (30 μg), and vancomycin (30 μg disk prior to 2010 then MIC by Etest [BioMerieux, France]). Nitrofurantoin (300 μg) is tested and reported in urine isolates only, while erythromycin and clindamycin are not reported in urine isolates. Our lab has continued to test for, and report, cefazolin and ceftriaxone susceptibility for MSSA isolates using the 2012 CLSI breakpoints [5]. According to CLSI recommendations, we measure the zone diameters (except for trimethoprim-sulfamethoxazole) by holding the Petri plate a few inches above a black background illuminated with reflected light, except for linezolid, which was read with transmitted light [11]. Weekly quality controls are performed using *S. aureus* ATCC 25923 for disk diffusion and *S. aureus* ATCC 29213 for Etest tested on Mueller–Hinton agar (Remel, USA) with acceptable in-range limits (Additional file 1: Figure S1). For this study, repeat isolates on the same patients within a calendar year were excluded, regardless of specimen source.

We retrospectively examined the cefazolin and ceftriaxone susceptibility profiles of all MSSA isolates in our lab between 2011 and 2016. To assess ceftriaxone use among patients with MSSA infections, we used records maintained by our Antimicrobial Stewardship Program (ASP) to identify a subset of patients that had MSSA-positive blood cultures and received ceftriaxone during this period. We then reviewed patient medical records to determine whether ceftriaxone was used as definitive therapy.

## Results and discussion

### General findings

**Fig. 1:**
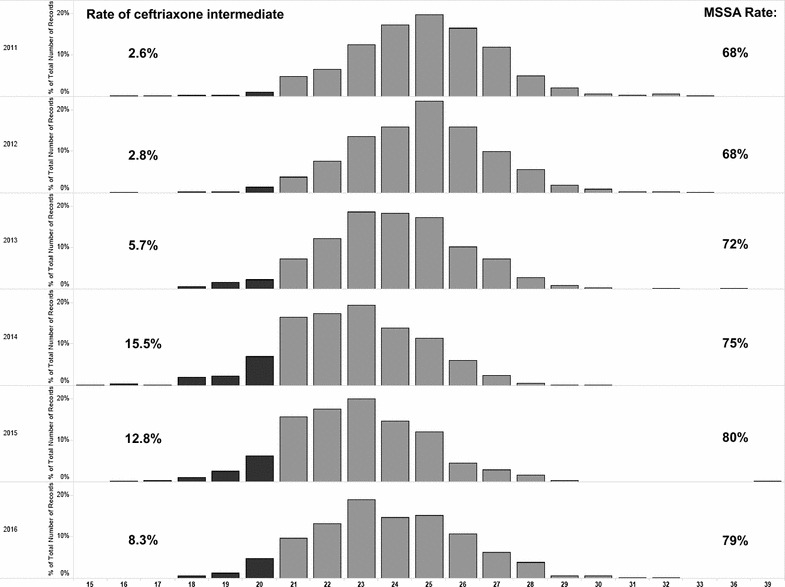
Ceftriaxone zone diameters (mm) for MSSA isolates, separated by year collected. Black bars are zone diameters in “Intermediate” range (14–20 mm), light gray bars are zone diameters in “Susceptible” range (≥ 21 mm). Values in top left corner of each pane reflect percent “intermediate” of all MSSA isolates to ceftriaxone in each year.  Values in top right corner of each pane reflect percent of *S. aureus *isolates that were methicillin susceptible (MSSA) in each year

A total of 3992 MSSA isolates tested between 2011 and 2016 were included in the susceptibility analysis. Using 2012 CLSI criteria, we confirmed that cefazolin remained an active agent, with all zone interpretive range confined above the “susceptible range of ≥ 19 mm (susceptible: ≥ 18 mm), and none in “intermediate” range of 15–17 mm (data not shown). We also found that a proportion of MSSA isolates produced ceftriaxone susceptibilities in “intermediate” zones ranges (14–20 mm) over the 6 year period, from 2.6% in 2011 to 8.3% in 2016 with the highest of 15.5% in 2014 (Fig. 1). None of the MSSA isolates produced ceftriaxone zone measurements in the “resistant” range (Fig. 1).

When ceftriaxone susceptibilities were further broken down by site of culture, we found that while blood, respiratory, and wound/deep tissue infections had similar proportion of MSSA isolates with ceftriaxone “intermediate” at 6.30, 6.82, and 6.15% respectively, the MSSA strains isolated from urine cultures showed higher rate of non-susceptibility to ceftriaxone (11.27%, Table 1). From the same period, methicillin-sensitive coagulase-negative *Staphylococcus* species (MS-CoNS, n = 673), were observed to be 8.0% in “intermediate” range to ceftriaxone. This fraction reduced to 3.1% when *S. saprophyticus* isolates (n = 80) were excluded, which accounted for the majority of MS-CoNS ceftriaxone non-susceptibility in our study. No “intermediate” range susceptibility to cefazolin was observed in MS-CoNS isolates, which was 100% sensitive over the course of our study (data not shown).

**Table 1:** Ratio of ceftriaxone susceptibilities against the 6-year MSSA isolates based on specimen source

Specimen type (group)	Ceftriaxone susceptibility	
	Intermediate (%)	Susceptible (%)
Blood (n = 349)	6.30	93.70
Cerebrospinal fluid (n = 22)		100.00
Osteoarticular (n = 33)	3.03	96.97
Respiratory tract (n = 1642)	6.82	93.18
Urine (n = 213)	11.27	88.73
Wound and deep tissue infections (n = 3348)	6.15	93.85

### In vitro susceptibility patterns

**Fig. 2:**
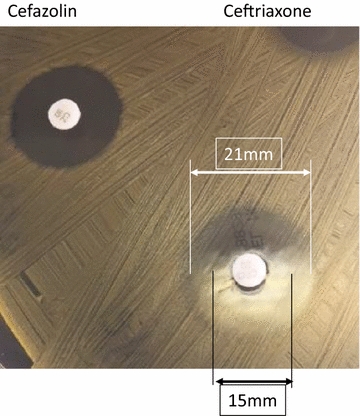
Characteristic “Cliff” versus “Beach” inhibitory zones associated with cefazolin and ceftriaxone respectively by disk diffusion method against MSSA isolates

Upon closer examination, growth patterns of MSSA isolates around ceftriaxone disks characteristically meeting the CLSI description of a “beach” type of heterogeneous inhibitory zone, as opposed to a “cliff” inhibitory zone around cefazolin (Fig. 2) [11]. Post hoc crosscheck of zone characteristics around of cefazolin and ceftriaxone disks on clinical-convenient samples (n = 153) have confirmed the distinct inhibitory zone characteristics between the two as shown in Additional file 1: Figure S2.

### Clinical practices and current state of in vitro testing

During the study period of 2011–2016, 321 patients with MSSA bacteremia were identified, in which only 6 patients received ceftriaxone as definitive therapy (after final susceptibility reports) without evidence of treatment failure.

Our institutional Antimicrobial Stewardship Committee has implemented a Microbiology Result Comment of “*β*-lactams like cefazolin and nafcillin are superior to vancomycin for treatment of MSSA” since mid-2013. This has, in part, resulted in our low number of cases where ceftriaxone was used as the definitive therapy. This hinders our ability to assess the clinical implications of ceftriaxone non-susceptibility (or “intermediate”), as all patients who received ceftriaxone as definitive therapy had known-susceptible isolates due to our cephalosporin susceptibility testing and reporting practices.

Ceftriaxone is not a first-line agent for MSSA infections, but its favorable dosing parameters often makes it a favorable choice in outpatient management. While there are no randomized controlled trials examining its effectiveness in treating MSSA infections, there have been small observational studies examining clinical outcomes, though it is not apparent that these studies tested for in vitro ceftriaxone non-susceptibility. One retrospective study from Israel investigated treatment of MSSA bacteremia in 541 patients with different *β*-lactams, using “inferred susceptibility” [9]. Ceftriaxone therapy was associated with higher 30-day adjusted mortality odds, though this did not hold with 90-day mortality. Another retrospective cohort study from Texas investigated ceftriaxone versus cefazolin therapy for invasive MSSA infections in 122 patients who received outpatient parenteral antibiotic therapy, finding similar clinical outcomes and adverse events [8]. Lastly, investigators from Missouri retrospectively compared ceftriaxone versus oxacillin in 124 patients with MSSA osteomyelitis and/or septic arthritis, finding similar clinical outcomes, though acknowledging fewer medication side effects with ceftriaxone treatment [10]. These studies highlight the need for further clinical trials.

The low historical prevalence of MSSA resistance to cefazolin, ceftriaxone and other antistaphylococcal β-lactams is well-described [12], and reflected in the recommendation to remove most β-lactam breakpoints from the 2013 CLSI (M100-S23) guidelines. As a result of this recommendation, cephalosporin agents such as cefazolin and ceftriaxone have been eliminated from most commercial antistaphylococcal susceptibility panels for surveillance information. Our data from continued testing of cefazolin and ceftriaxone using agar disk diffusion alone, without a precise MIC correlation clearly has limitations. It is possible there is a *mecA*-independent mechanism in MSSA and MS-CoNS conferring their reduced susceptibility to certain *β*-lactams, similar to the ever-emerging multitude of resistance in Gram-negative bacteria. This may need to be addressed for surveillance purposes. Interestingly, ceftriaxone non-susceptibility was not as pronounced in MS-CoNS, except for *S. saprophyticus*, which has a low overall prevalence of *mecA* positivity [13].

## Conclusions

This study affirms that cefazolin should continue to be the first line choice for the treatment of MSSA infections, and its inferred in vitro susceptibility from cefoxitin is still accurate. Our observed drift in ceftriaxone in vitro susceptibilities serves as an awareness call for closer surveillance. Additional studies at other institutions are necessary to determine if this trend is wide-spread. Consistent with CLSI recommendations, laboratory testing of antimicrobial susceptibility not only plays a role in patient care but also epidemiology surveillance for emergence of resistance.

## Additional file

**Additional file 1: Figure S1.** Quality control performances of weekly cefoxitin, cefazolin, and ceftriaxone disk diffusion the study period. **Figure S2.** Post hoc measurement of zones of inhibition generated from ceftriaxone and cefazolin disks against clinical isolates (n = 153) of MSSA. Heterogeneous or “beach”-type of zone phenotypes around ceftriaxone disk could be sized typically by either at ~ 80% growth inhibition or at the complete growth inhibition (Note: The measurement at the complete growth inhibition has been the standard for susceptibility interpretations). Insert graph shows “cliff”-type of homogeneous zone measurements around cefazolin disk.

## Abbreviations

MSSA: methicillin-susceptible *Staphylococcus aureus*
MIC: minimum inhibitory concentration
MS-CoNS: methicillin-sensitive coagulase-negative *Staphylococcus* species
ASP: Antimicrobial Stewardship Program
