# Supplementary material for: Continued in vitro cefazolin susceptibility in methicillin-susceptible Staphylococcus aureus
Source: Ann Clin Microbiol Antimicrob. 2018 Feb 20;17:5. doi: 10.1186/s12941-018-0257-x (PMC5819674; doi:10.1186/s12941-018-0257-x)

Figure S1. Quality control performances of weekly cefoxitin, cefazolin, and ceftriaxone disk diffusion during the study period.

Cefoxitin

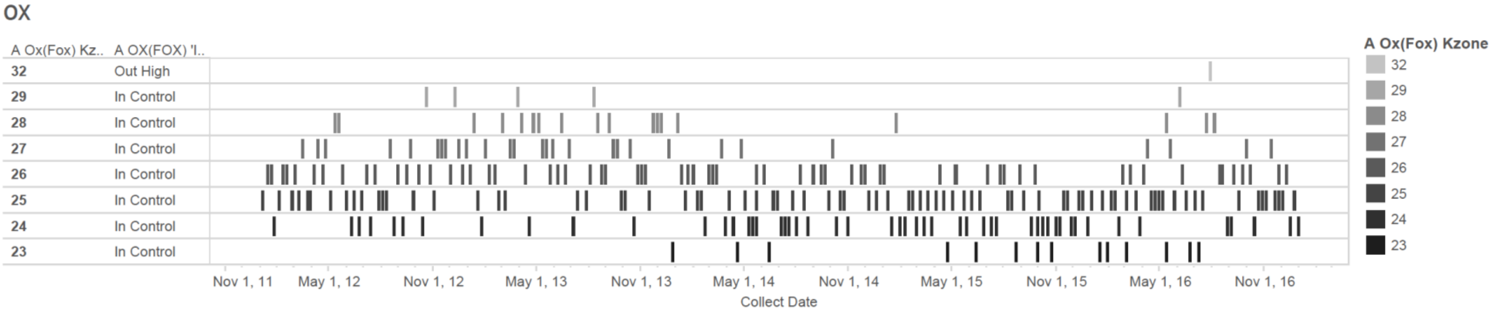

Cefazolin

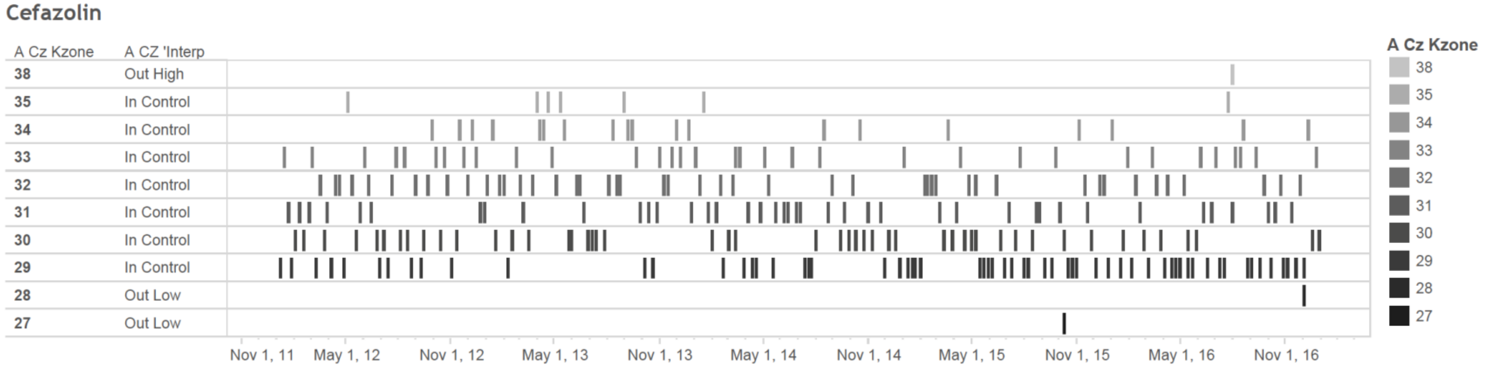

Ceftriaxone

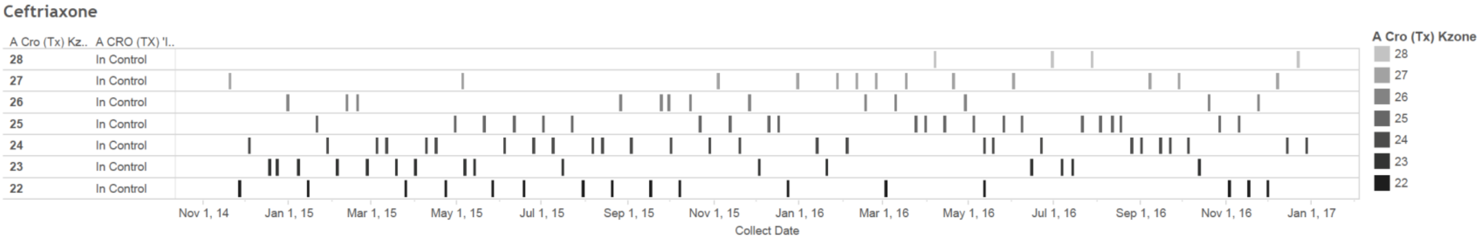

Figure S2. Post hoc measurement of zones of inhibition generated from ceftriaxone and cefazolin disks against clinical isolates (n=153) of MSSA. Heterogeneous or "beach"-type of zone phenotypes around ceftriaxone disk could be sized typically by either at ~80% growth inhibition or at the complete growth inhibition (Note: The measurement at the complete growth inhibition has been the standard for susceptibility interpretations). Insert graph shows "cliff"-type of homogeneous zone measurements around cefazolin disk.

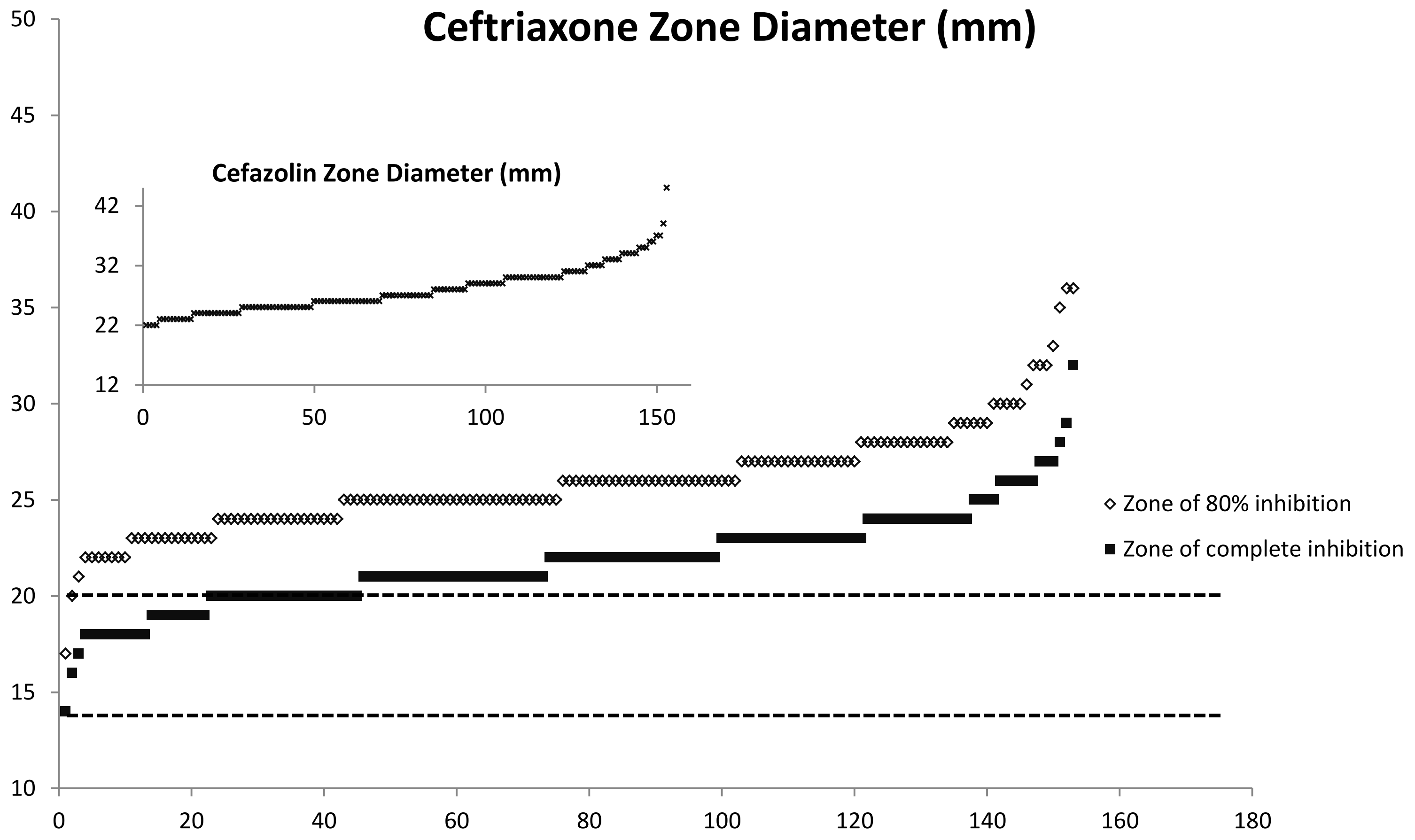

Supplement: Supplementary file 1 — Additional file 1: Figure S1. Quality control performances of weekly cefoxitin, cefazolin, and ceftriaxone disk diffusion the study period. Figure S2. Post hoc measurement of zones of inhibition generated from ceftriaxone and cefazolin disks against clinical isolates (n = 153) of MSSA. Heterogeneous or “beach”-type of zone phenotypes around ceftriaxone disk could be sized typically by either at ~ 80% growth inhibition or at the complete growth inhibition (Note: The measurement at the complete growth inhibition has been the standard for susceptibility interpretations). Insert graph shows “cliff”-type of homogeneous zone measurements around cefazolin disk. [file 12941_2018_257_MOESM1_ESM.pdf]
